# Chemotherapy as a critical treatment modality for non-surgical metaplastic breast cancer: evidence from SEER database and clinical cohort

**DOI:** 10.3389/fonc.2025.1534204

**Published:** 2025-05-12

**Authors:** Pin Wang, Huiying Fang, Dejuan Yang, Ying Liu, Ling Li, Jian Wu

**Affiliations:** ^1^ Department of General Surgery, The Third People’s Hospital of Chengdu, Chengdu, China; ^2^ Center of Breast and Thyroid Surgery, The Third People’s Hospital of Chengdu, Chengdu, China; ^3^ Department of Breast Cancer Center, Chongqing University Cancer Hospital, Chongqing, China; ^4^ Department of Breast and Thyroid Surgery, The First Affiliated Hospital of Chongqing Medical University, Chongqing, China; ^5^ Department of Ultrasound, The Third People’s Hospital of Chengdu, Chengdu, China; ^6^ Department of Pathology, The Third People’s Hospital of Chengdu, Chengdu, China

**Keywords:** metaplastic breast cancer, radiotherapy, chemotherapy, surgery, SEER

## Abstract

**Introduction:**

Metaplastic breast carcinoma (MPBC) is a highly aggressive subtype of breast cancer, characterized by enhanced metastatic potential and invasive behavior. While surgery is a cornerstone of treatment, there is limited research on patients who did not undergo surgery.

**Methods:**

We conducted a retrospective analysis of non-surgical MPBC patients using data from the Surveillance, Epidemiology, and End Results (SEER) database (1975–2019). Additionally, non-surgical MPBC patients were recruited from The Third People’s Hospital of Chengdu, The First Affiliated Hospital of Chongqing Medical University, and the Chongqing University Cancer Hospital to form the clinical validation cohort (2010–2024). We collected demographic and clinical data, including age, race, marital status, tumor location, and treatment modalities, etc. The primary endpoints were overall survival (OS) and breast cancer-specific survival (BCSS). Statistical analyses were performed using R software, with Kaplan-Meier curves and Cox regression models for univariate and multivariate analyses.

**Results:**

A total of 92 non-surgical MPBC patients were included from SEER database. The majority were aged≥60 years (65.22%), White (76.09%), and single (64.13%). Tumors were most frequently located in the upper quadrant (32.61%). Additionally, M1 patients were more likely to receive chemotherapy and radiotherapy compared to M0 patients (50.00% *vs*. 36.67%; 38.46% *vs*. 13.33%). Cox regression analysis identified chemotherapy and M stage as significant prognostic factors. Survival analysis showed that chemotherapy significantly improved OS and BCSS (*P*<0.001), while radiotherapy had no significant impact on survival (*P*>0.05). In the clinical cohort of 30 non-surgical MPBC patients, Kaplan-Meier curves demonstrated that chemotherapy significantly prolonged patient survival (*P*=0.039), whereas radiotherapy did not show a significant effect on survival (*P*=0.309).

**Conclusions:**

For MPBC patients who did not undergo surgery, chemotherapy significantly prolongs survival, highlighting its crucial role in treatment.

## 1 Introduction

Metaplastic breast cancer (MPBC) is a rare and highly aggressive subtype of breast cancer, characterized histologically by the transformation of neoplastic epithelium into cells with squamous or mesenchymal features (1). This process, known as metaplasia, results in the formation of abnormal tissue types within the breast that can behave aggressively and metastasize distant sites. Although MPBC accounts for only a small percentage of all breast cancers, it is disproportionately associated with a poorer prognosis compared to other breast cancer subtypes (2).

Clinically, MPBC is often managed similarly to triple-negative breast cancer (TNBC), given the lack of expression of hormone receptors and HER2 amplification (3). However, MPBC is generally more aggressive than TNBC, with higher rates of distant metastasis and worse overall prognosis. Due to its low incidence and significant heterogeneity, there are currently no specific treatment guidelines tailored to MPBC. Instead, treatment strategies are largely extrapolated from those used for TNBC and typically include surgery (4), chemotherapy (5), and radiotherapy (6).

The role of surgery in the treatment of MPBC has been increasingly recognized and validated (7). However, there remains a significant gap in research regarding treatment options for patients who did not undergo surgery due to factors such as refusal, tumor progression, comorbidities, or delayed diagnosis.

In this study, we aim to investigate the prognostic impact on MPBC patients who did not undergo surgical treatment. The data were obtained from the SEER database and from patients at The Third People’s Hospital of Chengdu, The First Affiliated Hospital of Chongqing Medical University, and the Chongqing University Cancer Hospital. We expect that this study will provide valuable insights into the management of MPBC patients who did not undergo surgical treatment.

## 2 Materials and methods

### 2.1 Data source and study design

**Figure 1:**
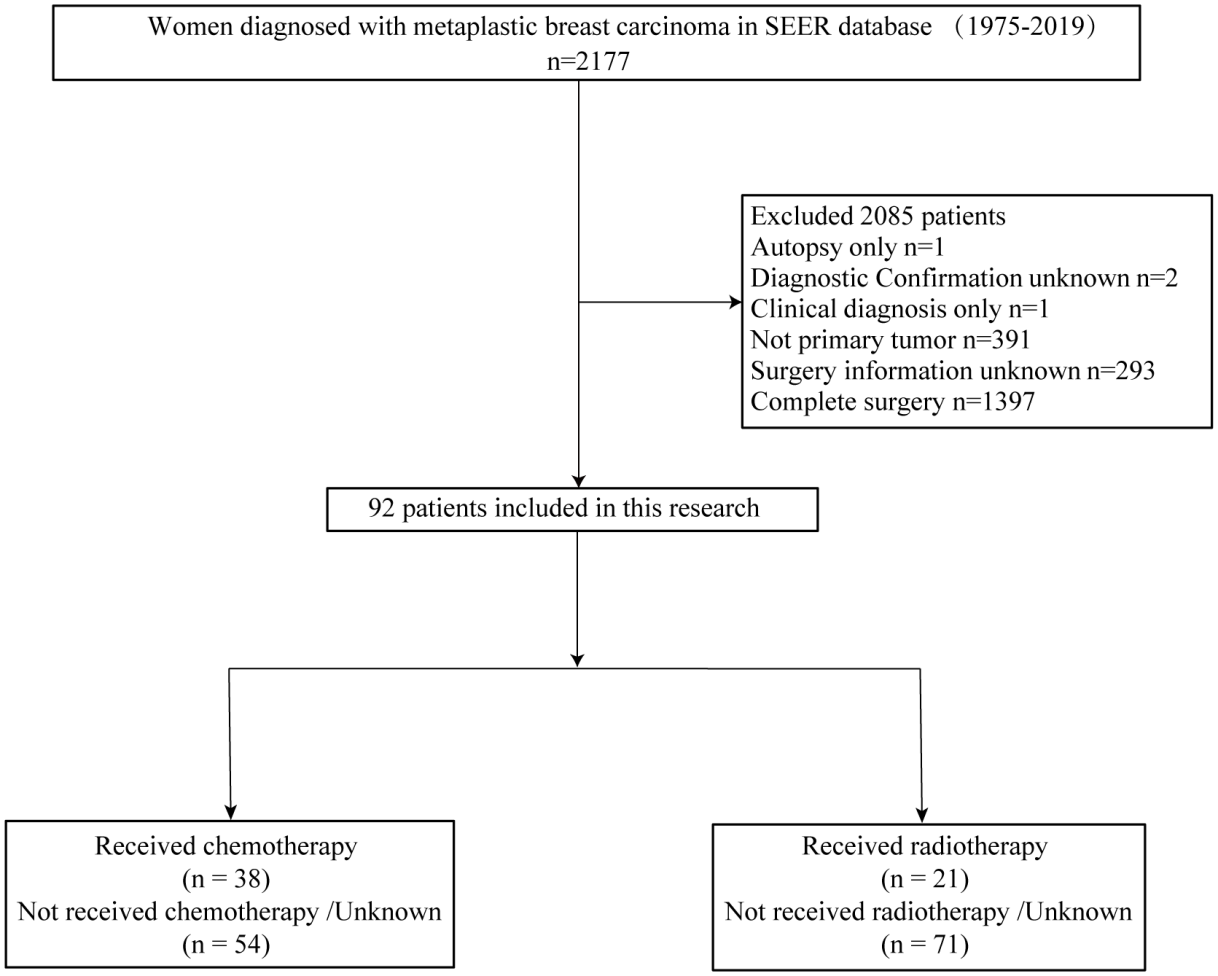
Flow chart: screening patients.

SEER*STAT 8.4.0.1 software was utilized to extract data from the SEER database for female patients diagnosed with MPBC in eight regions between 1975 and 2019. The entry criteria employed in the screening process included the following: (1) site recode ICD/WHO 2008 is breast, (2) patient is female, and (3) pathological diagnosis indicates metaplastic carcinoma based on ICD-0–3 morphology codes: 8032/3, 8035/3, 8052/3, 8070/3, 8071/3, 8072/3, 8073/3, 8074/3, 8075/3, 8560/3, 8562/3, 8570/3, 8571/3, 8572/3, 8573/3, 8575/3, 8980/3, or 8981/3. Exclusion criteria include: (1) inaccurate pathological diagnosis, (2) non-primary tumor status, and (3) unclear surgical information, (4) complete surgery. Ultimately, based on these inclusion and exclusion criteria, a total of 92 patients were selected for this study. **Figure 1** illustrates the patient selection process. Additionally, we collected data on 30 non-surgical MPBC patients from The Third People’s Hospital of Chengdu (7 cases), Chongqing University Cancer Hospital (8 cases), and The First Affiliated Hospital of Chongqing Medical University (15 cases) between 2010 and 2024.

### 2.2 Variables

The study incorporated the following demographic, baseline characteristics, and treatment information of the subjects: age, race, year of diagnosis, marital status, tumor site, grade, laterality, histological type, T, N, M stage, chemotherapy information, radiotherapy information, breast subtype, ER status, PR status, SEER cause-specific death classification, survival months, and vital status. As the HER2 variables in the SEER database were not available before 2010, most patients lacked records on HER2 status. The primary endpoints of the study were overall survival (OS) and breast cancer-specific survival (BCSS). OS was defined as the time from diagnosis to death from any cause, regardless of whether it was health related. BCSS was measured from the time of diagnosis until death specifically attributed to breast cancer.

### 2.3 Statistical analysis

For statistical analysis, R software (version 4.2.1) was used in this study. Kaplan–Meier survival curves were plotted using the survival package, and univariate and multivariate analyses were performed using Cox regression models from the same package. Variables with *P*-values <0.05 in univariate analysis were chosen for multivariate regression. The “forestplot” package was employed to construct forest plots.

## 3 Results

### 3.1 MPBC patients’ baseline characteristics

This retrospective study conducted a comprehensive analysis of 2177 patients diagnosed with MPBC from the SEER database, covering the period from 1975 to 2019. After applying inclusion and exclusion criteria, our final cohort consisted of 92 non-surgical patients.

**Table 1** provides an overview of the demographic and clinicopathological characteristics of 92 patients. Most of the cohorts were over 60 years old (65.22%) and White (76.09%). There was a notable increase in diagnoses in the last decade, with 67.39% of cases diagnosed between 2010 and 2019. A significant proportion of the group was single, accounting for 64.13%. Most common tumor location was the upper quadrant, affecting 32.61% of patients.

**Table 1:** Clinicopathological characteristics baseline (SEER database).

Characteristics	N= 92
Age	
20–39 years	2 (2.17%)
40–59 years	30 (32.61%)
>60 years	60 (65.22%)
Race	
Black	15 (16.30%)
Other	6 (6.52%)
White	70 (76.09%)
Unknown	1 (1.09%)
Year of diagnosis	
1975-2009	30 (32.61%)
2010-2019	62 (67.39%)
Marital status	
Married	27 (29.34%)
Single	59 (64.13%)
Unknown	6 (6.52%)
Tumor site	
Upper quadrant	30 (32.61%)
Lower quadrant	9 (9.78%)
Overlapping lesion	23 (25.00%)
Central portion	6 (6.52%)
Breast, NOS	24 (26.09%)
Grade*	
I	3 (3.26%)
II	8 (8.70%)
III	31 (33.70%)
IV	5 (5.43%)
Unknown	45 (48.91%)
Laterality	
Left	51 (55.43%)
Right	39 (42.40%)
Unknown	2 (2.17%)
Histological type	
Adenocarcinoma	9 (9.78%)
Carcinosarcoma	3 (3.26%)
Metaplastic carcinoma, NOS	57 (61.96%)
Spindle cell carcinoma	7 (7.61%)
Squamous cell carcinoma	16 (17.39%)
T stage	
T1	8 (8.70%)
T2	10 (10.87%)
T3	15 (16.30%)
T4	13 (14.13%)
Unknown	46 (50.00%)
N stage	
N0	27 (29.35%)
N1	18 (19.57%)
N2	7 (7.61%)
N3	8 (8.70%)
Unknown	32 (34.78%)
M stage	
M0	60 (65.22%)
M1	26 (28.26%)
Unknown	6 (6.52%)
Radiotherapy	
No/unknown	71 (77.17%)
Yes	21 (22.83%)
Chemotherapy	
No/unknown	54 (58.70%)
Yes	38 (41.30%)
Breast subtype (2010+)	
HR+/HER2+	4 (4.35%)
HR+/HER2-	10 (10.87%)
HR-/HER2+	2 (2.17%)
HR-/HER2-	35 (38.04%)
Unknown	41 (44.57%)
ER status	
Negative	58 (63.04%)
Positive	17 (18.48%)
Unknown	17 (18.48%)
PR status	
Negative	62 (67.39%)
Positive	9 (9.78%)
Unknown	21 (22.83%)

*Grade: I: well differentiated; II: moderately differentiated; III: poorly differentiated; IV: undifferentiated.

Regarding tumor staging, the distribution was as follows: 8 patients were classified as T1 (8.70%), 10 as T2 (10.87%), 15 as T3 (16.30%), and 13 as T4 (14.13%). In terms of lymph node involvement, 27 cases were N0 (29.35%), 18 were N1 (19.57%), 7 were N2 (7.61%), and 8 were N3 (8.70%), with 32 cases (34.78%) having an unknown N stage. For distant metastasis, 60 cases were M0 (65.22%), 26 were M1 (28.26%), and 6 cases (6.52%) had an unknown M stage.

**Figure 2:**
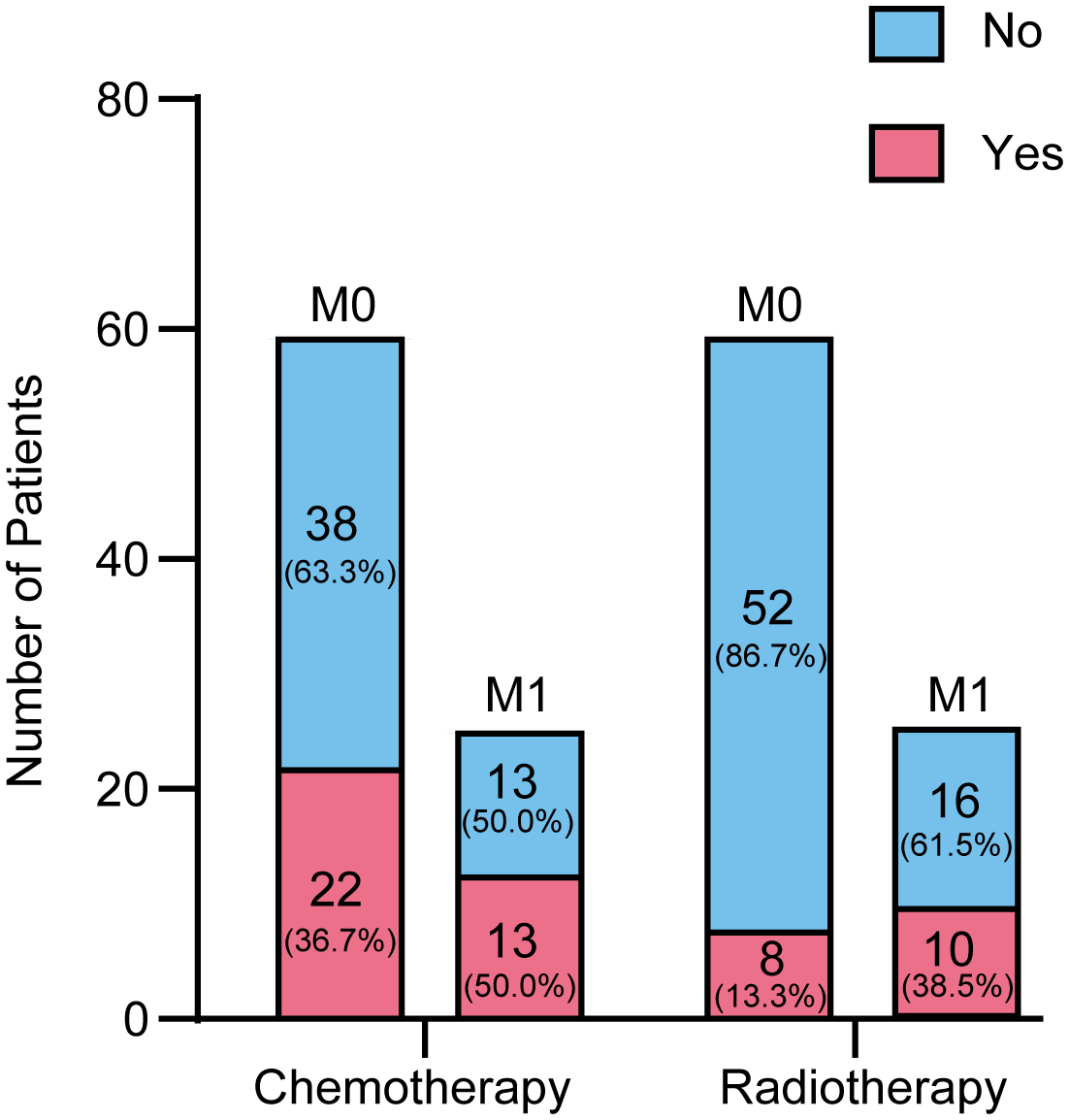
Chemotherapy and radiotherapy treatment rates among MPBC patients.

**Figure 2** provides a detailed overview of the distribution of patients undergoing chemotherapy and radiotherapy across different metastatic stages (M0 and M1). The data reveals that patients at the M1 stage are more likely to receive chemotherapy compared to those at the M0 stage (50% versus 36.67%). Specifically, out of 60 patients with M0 disease, 22 (36.67%) received chemotherapy. Among the 26 patients with M1 disease, 13 (50%) underwent chemotherapy. Regarding radiotherapy, 8 patients (13.33%) were treated, compared to 52 (86.67%) who were not. Among M1 patients, 10 (38.46%) received radiotherapy, while 16 did not. Additionally, the metastatic status of 6 patients was unknown, and thus they were not categorized under either M0 or M1. These figures indicate that patients with M1 disease tend to receive chemotherapy and radiotherapy, whereas these treatments are less common among M0 patients.

### 3.2 Prognostic factors for non-surgery MPBC patients

**Figure 3:**
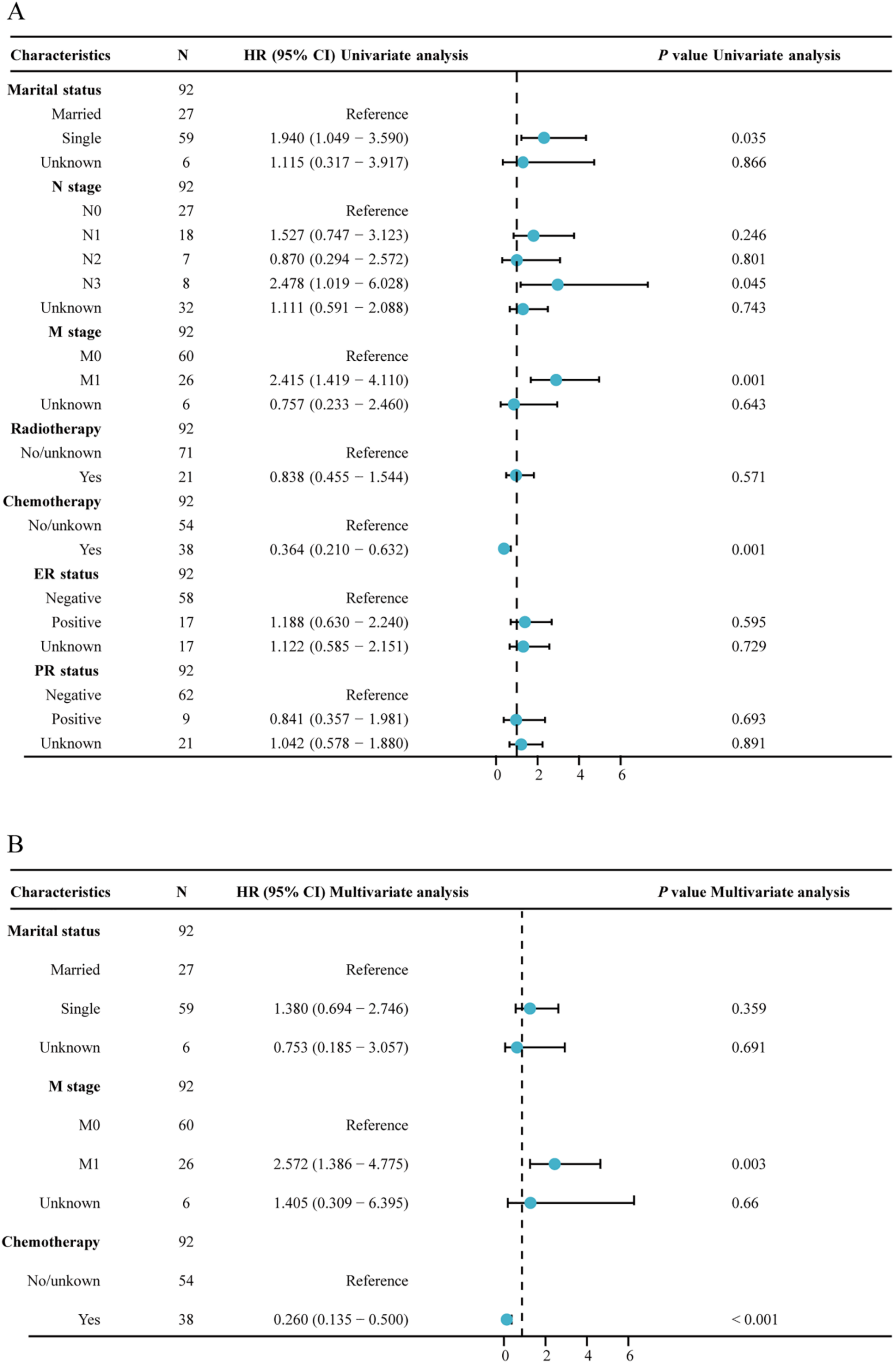
Forest plot of univariate and multivariate regression analysis. **(A)** univariate regression analysis; **(B)** multivariate regression analysis.

In our analysis of factors affecting survival in MPBC patients, several key prognostic factors emerged (**Table 2**; **Figure 3**). Patients at stage M1 exhibited a significantly higher risk in both univariate and multivariate analyses (HR = 2.415, *P* = 0.001; HR = 2.572, *P* = 0.003). Additionally, chemotherapy was a significant factor affecting survival rates; patients who received chemotherapy showed significantly lower risks in both univariate and multivariate analyses (HR = 0.364, *P* < 0.001; HR = 0.260, *P* < 0.001).

**Table 2:** Univariable and multivariable Cox proportional hazard regression model of overall survival (OS).

Characteristics	N=92	Univariate analysis HR (95% CI)	*P* value	Multivariate analysis HR (95% CI)	*P* value
Age					
>60 years	60	Reference		Reference	
40–59 years	30	0.533 (0.298 - 0.955)	0.034	0.663 (0.344 - 1.275)	0.218
20–39 years	2	0.000 (0.000 - Inf)	0.995	0.000 (0.000 - Inf)	0.996
Race					
White	70	Reference			
Black	15	0.680 (0.345 - 1.342)	0.266		
Other	6	1.519 (0.463 - 4.980)	0.490		
Unknown	1	0.000 (0.000 - Inf)	0.996		
Year of diagnosis					
1975-2009	30	Reference			
2010-2019	62	0.808 (0.485 - 1.345)	0.412		
Marital status					
Married	27	Reference		Reference	
Single	59	1.940 (1.049 - 3.590)	0.035	1.380 (0.694 - 2.746)	0.359
Unknown	6	1.115 (0.317 - 3.917)	0.866	0.753 (0.185 - 3.057)	0.691
Tumor site					
Upper quadrant	30	Reference			
Lower quadrant	9	1.328 (0.579 - 3.044)	0.503		
Overlapping lesion	23	1.413 (0.739 - 2.702)	0.296		
Central portion	6	0.827 (0.281 - 2.434)	0.731		
Breast, NOS	24	0.762 (0.382 - 1.521)	0.44		
Grade					
I	3	Reference			
II	8	0.353 (0.086 - 1.444)	0.147		
III	31	0.552 (0.164 - 1.862)	0.338		
IV	5	0.508 (0.111 - 2.315)	0.381		
Unknown	45	0.386 (0.115 - 1.300)	0.124		
Laterality					
Right	39	Reference			
Left	51	1.013 (0.611 - 1.680)	0.960		
Unknown	2	1.477 (0.348 - 6.259)	0.597		
Histological type					
Squamous cell carcinoma	16	Reference		Reference	
Adenocarcinoma	9	2.757 (1.103 - 6.892)	0.030	5.014 (1.620 - 15.519)	0.005
Metaplastic carcinoma, NOS	57	1.381 (0.687 - 2.773)	0.365	2.247 (0.969 - 5.210)	0.059
Spindle cell carcinoma	7	2.664 (0.827 - 8.579)	0.101	6.739 (1.860 - 24.410)	0.004
Carcinosarcoma	3	0.348 (0.044 - 2.725)	0.315	1.860 (0.134 - 25.732)	0.643
T stage					
T1	8	Reference		Reference	
T2	10	2.058 (0.247 - 17.150)	0.505	1.461 (0.173 - 12.350)	0.728
T3	15	5.999 (0.785 - 45.824)	0.084	5.257 (0.663 - 41.703)	0.116
T4	13	4.878 (0.614 - 38.759)	0.134	3.659 (0.424 - 31.566)	0.238
Unknown	46	2.780 (0.378 - 20.427)	0.315	2.848 (0.358 - 22.679)	0.323
N stage					
N0	27	Reference			
N1	18	1.527 (0.747 - 3.123)	0.246		
N2	7	0.870 (0.294 - 2.572)	0.801		
N3	8	2.478 (1.019 - 6.028)	0.045		
Unknown	32	1.111 (0.591 - 2.088)	0.743		
M stage					
M0	60	Reference		Reference	
M1	26	2.415 (1.419 - 4.110)	0.001	2.572 (1.386 - 4.775)	0.003
Unknown	6	0.757 (0.233 - 2.460)	0.643	1.405 (0.309 - 6.395)	0.66
Radiotherapy					
None/Unknown	71	Reference			
Yes	21	0.838 (0.455 - 1.544)	0.571		
Chemotherapy					
No/Unknown	54	Reference		Reference	
Yes	38	0.364 (0.210 - 0.632)	< 0.001	0.260 (0.135 - 0.500)	< 0.001
Breast subtype (2010+)					
HR-/HER2-	35	Reference			
HR+/HER2+	4	0.483 (0.113 - 2.056)	0.324		
HR+/HER2-	10	1.594 (0.677 - 3.755)	0.286		
HR-/HER2+	2	2.717 (0.625 - 11.810)	0.183		
Unknown	41	1.158 (0.667 - 2.008)	0.602		
ER status					
Negative	58	Reference			
Positive	17	1.188 (0.630 - 2.240)	0.595		
Unknown	17	1.122 (0.585 - 2.151)	0.729		
PR status					
Negative	62	Reference			
Positive	9	0.841 (0.357 - 1.981)	0.693		
Unknown	21	1.042 (0.578 - 1.880)	0.891		

In contrast, age, marital status, tumor location, tumor grade, laterality, histological type, T stage, N stage, radiotherapy and ER and PR status did not significantly impact survival rates. These results indicate that, among MPBC patients, chemotherapy and M stage are important prognostic factors affecting survival rates.

### 3.3 Chemotherapy extends survival in non-surgery MPBC patients

**Figure 4:**
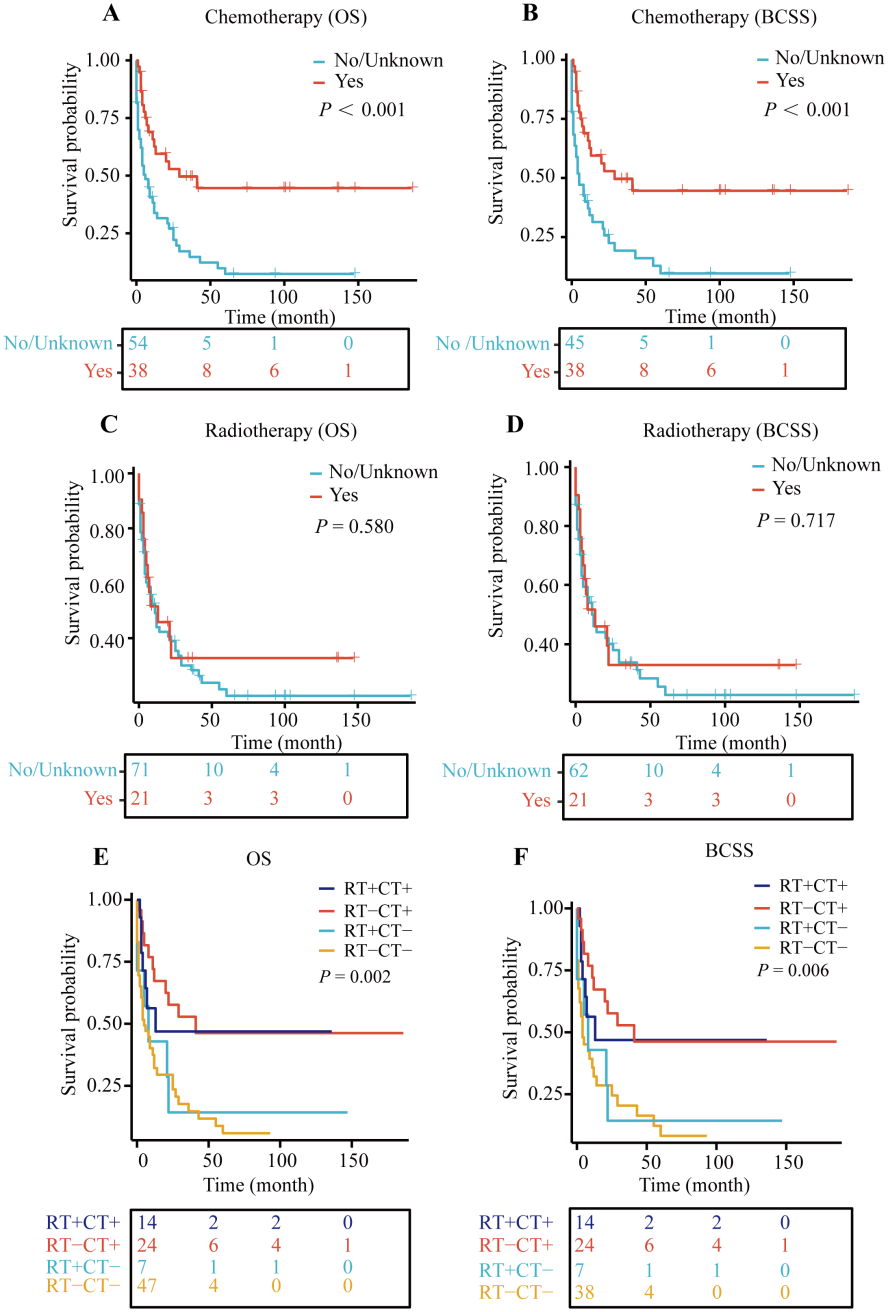
Kaplan–Meier (KM) curves for OS and BCSS. **(A)** KM curve for OS in chemotherapy and non-chemotherapy group, **(B)** KM curve for BCSS in chemotherapy and non-chemotherapy group, **(C)** KM curve for OS in radiotherapy and non-radiotherapy group, **(D)** KM curve for BCSS in radiotherapy and non-radiotherapy group, **(E)** Kaplan-Meier curve for OS in patients treated with radiotherapy (RT) and/or chemotherapy (CT), **(F)** Kaplan-Meier curve for BCSS in patients treated with radiotherapy (RT) and/or chemotherapy (CT).

Patients were divided into groups based on whether they received chemotherapy and radiotherapy. The Kaplan-Meier survival curves indicated that patients who underwent chemotherapy experienced a significant improvement in both overall survival (OS) and breast cancer-specific survival (BCSS) compared to those who did not, with both survival rates showing *P*-values less than 0.001 (**Figures 4A, B**).

However, when we analyzed the survival outcomes of patients who received radiotherapy versus those who did not, no significant differences in survival were observed (**Figures 4C, D**).

Subsequently, we examined the survival rates of patients who received both chemotherapy and radiotherapy. The findings revealed that regardless of radiotherapy, patients who received chemotherapy had better prognoses than those who did not (with OS *P*-value of 0.002 and BCSS *P*-value of 0.006) (**Figures 4E, F**). This suggests that, among patients not undergoing surgery, chemotherapy alone may enhance survival outcomes to some extent.

### 3.4 Chemotherapy extends survival in non-surgical MPBC patients in clinical cohort

**Figure 5:**
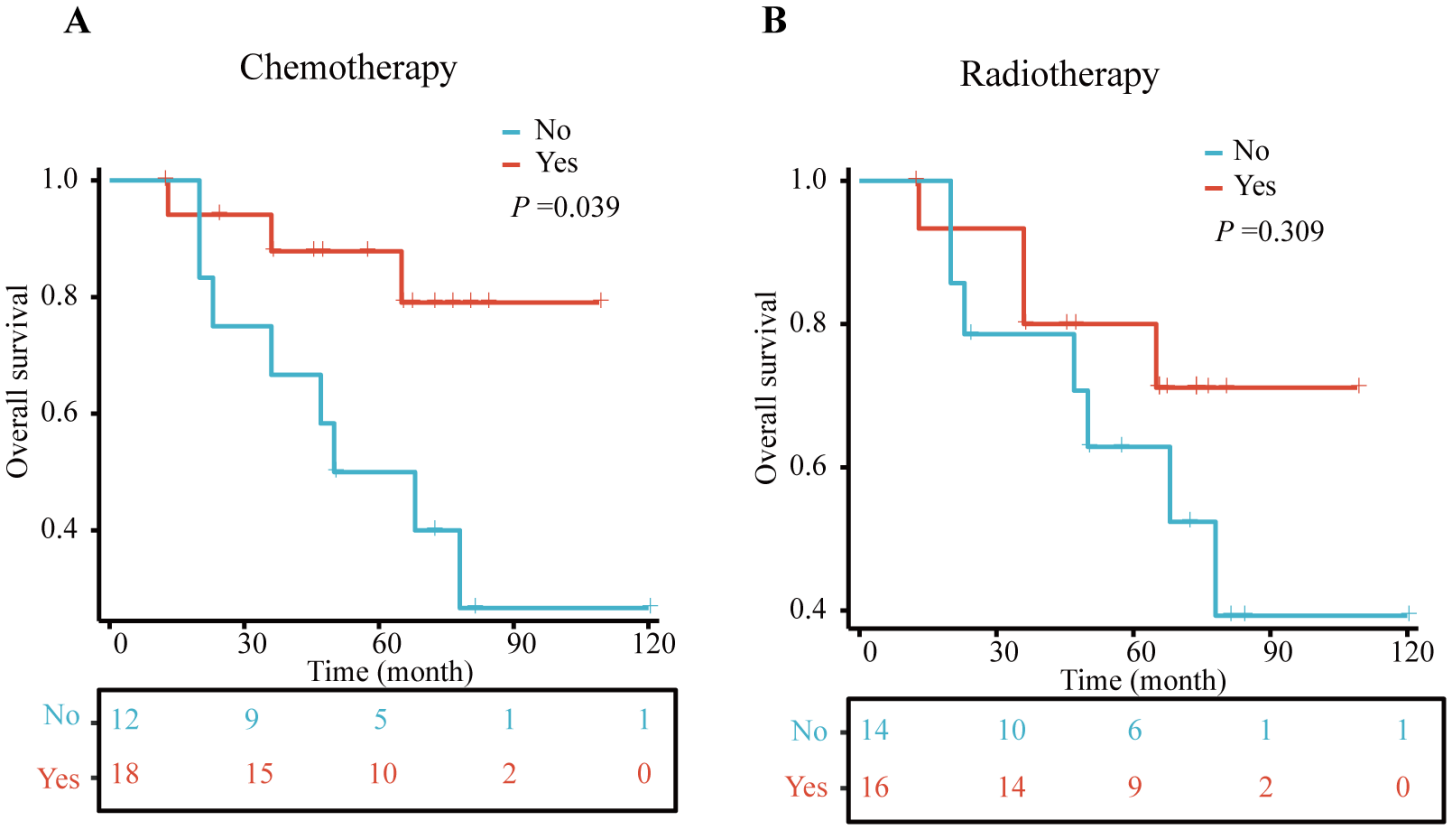
Kaplan–Meier (KM) curves for OS in clinical cohort. **(A)** chemotherapy and non-chemotherapy group, **(B)** radiotherapy and non- radiotherapy group.

Between 2010 and 2024, data were collected from 30 non-surgical MPBC patients treated at The Third People’s Hospital of Chengdu (7 cases), Chongqing University Cancer Hospital (8 cases), and The First Affiliated Hospital of Chongqing Medical University (15 cases). The mean age was 53.97 ± 10.97 years, with 53.33% of patients being single. Tumors were evenly distributed between the left and right breasts (50% each). Histologically, metaplastic carcinoma was the most common subtype (60%), Tumor staging included T1-T4, with T3 being the most frequent (40%). Most patients had regional lymph node involvement (N1-3, 70%), and over half had distant metastasis (M1, 53.33%). Receptor status showed high rates of ER negativity (86.67%) and PR negativity (100.00%) (**Table 3**). Chemotherapy was administered to 60% of patients, and radiotherapy to 53.33%. Kaplan-Meier analysis revealed that chemotherapy significantly improved overall survival (*p* = 0.039), while radiotherapy did not show a significant survival benefit (*p* = 0.309) (**Figure 5**).

**Table 3:** Clinicopathological characteristics baseline (clinical cohort).

Characteristics	N= 30
Age	53.97 ± 10.97
Marital status	
Married	14 (46.67%)
Single	16 (53.33%)
Laterality, n (%)	
Left	15 (50.00%)
Right	15 (50.00%)
Histological type	
Metaplastic carcinoma	18 (60.00%)
Spindle cell carcinoma	3 (10.00%)
Carcinosarcoma	3 (10.00%)
Adenocarcinoma	2 (6.67%)
Metaplastic carcinoma with osseous differentiation	3 (10.00%)
Squamous cell carcinoma	1 (3.33%)
T stage	
T1	4 (13.33%)
T2	9 (30.00%)
T3	12 (40.00%)
T4	5 (16.67%)
N stage	
N0	9 (30.00%)
N1	6 (20.00%)
N2	8 (26.67%)
N3	7 (23.33%)
M stage	
M0	14 (46.67%)
M1	16 (53.33%)
ER status	
Negative	26 (86.67%)
Positive	4 (13.33%)
PR status	
Negative	30 (100.00%)
Positive	0 (0.00%)
Her-2 status	
0	23 (76.67%)
1+	6 (20.00%)
2+	0 (0.00%)
3+	1 (3.33%)
Chemotherapy	
Yes	18 (60.00%)
No	12 (40.00%)
Radiotherapy	
Yes	16 (53.33%)
No	14 (46.67%)

## 4 Discussion

MPBC is a rare and highly aggressive subtype of breast cancer, characterized by its heterogeneous histology and poor prognosis (8). While surgery remains the cornerstone of treatment for MPBC, a portion of patients are not undergoing surgical intervention due to advanced disease, comorbidities, or patient refusal. This study aimed to evaluate the prognostic impact of chemotherapy and radiotherapy in non-surgical MPBC patients using both data from the SEER database and clinical cohort. Our findings demonstrate that chemotherapy significantly improves overall survival (OS) and breast cancer-specific survival (BCSS) in this patient population, while radiotherapy did not show a significant survival benefit. These results underscore the critical role of chemotherapy as a systemic treatment option for non-surgical MPBC patients.

The significant survival benefit observed with chemotherapy in non-surgical MPBC patients has highlighted the aggressive nature of this cancer subtype. MPBC is often compared to triple-negative breast cancer (TNBC) due to its lack of hormone receptor and HER2 expression, which limits the effectiveness of endocrine and targeted therapies (9). In our clinical cohort, the rates of estrogen receptor (ER) and progesterone receptor (PR) negativity were significantly higher than those reported in the SEER database (86.7% and 100%, respectively). Chemotherapy, therefore, remains one of the few systemic treatment options available for MPBC patients. Previous research has indicated that chemotherapy may exert its therapeutic effects in MPBC patients by inhibiting epithelial-mesenchymal transition (EMT) processes (10). However, the SEER database lacks detailed information on specific chemotherapy regimens, limiting our ability to assess the efficacy of different drug combinations or dosing strategies. Future studies should aim to identify optimal chemotherapy protocols for MPBC, particularly in the non-surgical setting.

In our clinical cohort, the proportion of patients with M1 disease was higher than that in the SEER database cohort (53.3% *vs*. 26.28%). Although radiotherapy did not significantly impact survival in our study, its role in palliative care for advanced MPBC patients should not be overlooked. Radiotherapy is often used to manage local symptoms, such as pain from bone metastases or neurological symptoms from brain metastases. While it may not extend survival, radiotherapy can improve quality of life by alleviating debilitating symptoms. This is particularly relevant for non-surgical patients, who may benefit from symptom control. Future research should explore the impact of radiotherapy on patient-reported outcomes, such as pain relief and functional status, to better understand its role in the management of MPBC.

Current studies on MPBC predominantly concentrate on evaluating the efficacy of chemotherapy and radiotherapy, while lacking comprehensive stratification based on surgical intervention status. For example, Chen et al. demonstrated that chemotherapy might improve survival outcomes in patients with T1c tumors but not in those with T1a or T1b tumors (11). Li et al. observed that radiotherapy may be advantageous for elderly patients or those with larger tumors (12). Wang et al. showed that radiotherapy could enhance breast cancer-specific survival in high-risk patients (13). Hu et al. proposed that postoperative radiotherapy for metaplastic breast cancer could improve breast cancer-specific survival in high-risk groups and overall survival in medium- to high-risk groups (14). Although previous studies have demonstrated the benefits of radiotherapy in reducing local recurrence and improving survival in surgical patients, our results suggest that these benefits may not apply to non-surgical patients, as they often present with more advanced diseases. This discrepancy calls for a more nuanced approach to MPBC treatment, particularly in non-surgical settings, where systemic therapies such as chemotherapy may play a more critical role.

MPBC is characterized by a high degree of molecular heterogeneity, with frequent mutations in genes. These molecular alterations may contribute to the aggressive behavior of MPBC and its resistance to conventional therapies (15). One study reported a case of MPBC with a BRAF V600E mutation that showed a positive response to targeted therapy with dabrafenib and trametinib, highlighting the potential for personalized treatment methods in MPBC (16). Another study demonstrated that MPBC patients had an excellent response to combination therapy including PD-L1 inhibitors and paclitaxel (17, 18). Common genetic mutations in MPBC, such as TP53, PIK3CA, and PTEN, suggest that new targeted strategies could improve survival outcomes for patients (15). A recent study showed that mTOR inhibitors can extend survival in patients with advanced breast cancer or metaplastic triple-negative breast cancer, indicating that drugs targeting the PI3K/AKT/mTOR pathway may benefit MPBC patients (19). Additionally, methylation of the BRCA1 gene promoter has been identified as a potentially relevant factor in metaplastic cancers (20). Research also indicates that activation of the WNT signaling pathway and amplification, and overexpression of the EGFR gene frequently occur in MPBC, pointing to potential therapeutic targets (21, 22). While the treatments show promises for MPBC, all require further validation through large-scale clinical trials.

Our study has several limitations that should be acknowledged. First, the SEER database lacks detailed information on chemotherapy regimens, radiotherapy doses, and patient comorbidities, which may influence treatment outcomes. Second, the small sample size of non-surgical MPBC patients limits the generalizability of our findings. Larger, prospective studies are needed to validate these results and explore the impact of different treatment modalities in this patient population. Additionally, the retrospective nature of our study introduces potential selection bias, as patients who received chemotherapy may have had better performance status or fewer comorbidities. Future studies should aim to control these confounding factors through propensity score matching.

In conclusion, our study demonstrates that chemotherapy significantly improves survival in non-surgical MPBC patients, highlighting its importance as a systemic treatment option. While radiotherapy did not show a survival benefit, it remains a valuable tool for symptom control local symptoms. Future research should focus on identifying optimal chemotherapy regimens, exploring the role of targeted therapies and immunotherapy, and improving palliative care for non-surgical MPBC patients. By addressing these gaps, we can aim to improve outcomes for this challenging and understudied non-surgery patient population.

## Data Availability

The raw data supporting the conclusions of this article will be made available by the authors, without undue reservation.
